# Autonomy in Depressive Patients Undergoing DBS-Treatment: Informed Consent, Freedom of Will and DBS’ Potential to Restore It

**DOI:** 10.3389/fnint.2017.00011

**Published:** 2017-06-08

**Authors:** Timo Beeker, Thomas E. Schlaepfer, Volker A. Coenen

**Affiliations:** ^1^Department of Psychiatry and Psychotherapy, Medical School Brandenburg Theodor FontaneRüdersdorf, Germany; ^2^Department of Interventional Biological Psychiatry, Freiburg University Medical CenterFreiburg, Germany; ^3^Medical Faculty, Freiburg UniversityFreiburg, Germany; ^4^Department of Stereotactic and Functional Neurosurgery, Freiburg University Medical CenterFreiburg, Germany

**Keywords:** deep brain stimulation, depression, autonomy, informed consent, decision making, neuromodulation, neuroethics

## Abstract

According to the World Health Organization, depression is one of the most common and most disabling psychiatric disorders, affecting at any given time approximately 325 million people worldwide. As there is strong evidence that depressive disorders are associated with a dynamic dysregulation of neural circuits involved in emotional processing, recently several attempts have been made to intervene directly in these circuits via deep brain stimulation (DBS) in patients with treatment-resistant major depressive disorder (MDD). Given the promising results of most of these studies, the rising medical interest in this new treatment correlates with a growing sensitivity to ethical questions. One of the most crucial concerns is that DBS might interfere with patients’ ability to make autonomous decisions. Thus, the goal of this article is to evaluate the impact DBS presumably has on the capacity to decide and act autonomously in patients with MDD in the light of the autonomy-undermining effects depression has itself. Following the chronological order of the procedure, special attention will first be paid to depression’s effects on patients’ capacity to make use of their free will in giving valid Informed Consent. We suggest that while the majority of patients with MDD appear capable of autonomous choices, as it is required for Informed Consent, they might still be unable to effectively act according to their own will whenever acting includes significant personal effort. In reducing disabling depressive symptoms like anhedonia and decrease of energy, DBS for treatment resistant MDD thus rather seems to be an opportunity to substantially increase autonomy than a threat to it.

## Introduction

The introduction of the minimally invasive and highly precise stereotactic method, which is also the basis of deep brain stimulation (DBS), has most certainly been helped by the desire to overcome the often gruesome practice of frontal lobotomy (Gildenberg and Krauss, 2009). In 1947, the American neurologist Ernest A. Spiegel and neurosurgeon Henry T. Wycis were the first to use the stereotactic apparatus on humans and described dorsomedial thalamotomy for depression and anxiety disorders (Gildenberg, 2002,^1^). Due to its tendency to induce cognitive deficits, the dorsomedial thalamus was soon replaced by other target regions for lesion surgery that have just recently been investigated for their distant connectivity (Schoene-Bake et al., 2010). Lozano and Mayberg first applied DBS technology on treatment resistant major depressive disorder (MDD) with a formerly unknown efficacy in 2005 (Mayberg et al., 2005). Over the years, other target regions were tested (see, Table 1), all with strikingly similar anti-depressant effects^1^. Whereas one recently published multicenter study on DBS of the Ventral Capsule/Ventral Striatum failed to reproduce the remission rates of the initial studies (Dougherty et al., 2015), a pilot study using the superolateral medial forebrain bundle (slMFB) as new target reported heretofore never achieved anti-depressant efficacy of 85%. Remarkably, this latter target has been developed in a hypothesis-guided way (Coenen et al., 2011). Stimulation of this region, which is until today mostly known for its role in motivated behavior and addiction research (Panksepp, 1998), required even significantly less energy than for all previous targets (Schlaepfer et al., 2014).

**Table 1:** Previous studies on deep brain stimulation (DBS) for major depressive disorder (MDD) with three or more participants (Original table).

Study	Target structure	Patients treated	Results
Lozano et al. (2008)	Subgenuale cingulate gyrus (Brodmann-Areal 25, Cg25)	20	6 months follow up: response^a^ 12/20, remission 7/20^b^
Malone et al. (2009)	Anterior limb of internal capsule (ALIC)	15	6 months follow up: response 7/15, remission 3/15
Schlaepfer et al. (2008)	Accumbens nucleus (NAC)	3	6–23 weeks follow up: response 1/3
Bewernick et al. (2010)	NAC	10	12 months follow up: response 5/10
Holtzheimer et al. (2012)^c^	Subcallosal Cingulate Gyrus (SCG)	17	2 years follow up: response 11/12, remission 7/12
Puigdemont et al. (2012)	SCG	8	12 months follow up: 5/8 response, remission 4/8
Merkl et al. (2013)	SCG	6	24–36 weeks follow up: 2/6 remission
Ramasubbu et al. (2013)	SCG	4	6 months follow up: response 2/4
Schlaepfer et al. (2014)	Superolateral branch of the medial forebrain bundle (slMFB)	7	up to 6 months follow up; 6/7 response^d^
Dougherty et al. (2015)	Ventral Capsule/Ventral Striatum	15	3/15 response within 16 weeks
Accolla et al. (2016)	Posterior gyrus rectus region/(Cg 25)	1/(4)^e^	1/1 response; (0/4 response)
Bergfeld et al. (2016)	ALIC	25	12 months follow up: response 10/25
Fenoy et al. (2016)	slMFB	4	26 weeks follow up: response 2/3^f^

^a^Response is commonly defined as a reduction of more than 50% of baseline depressive symptoms, measured either with Hamilton Rating Scale for Depression (HRSD) or Montgomery-Åsberg Depression Rating Scale (MADRS).

^b^Every Patient in remission counts at the same time as a responder. Thus remission and response-numbers cannot simply be added.

^c^In addition to 10 patients with MDD, seven patients with bipolar II Disorder were enrolled.

^d^In a recently published long time observation over the course of 4 years including one more patient, a stable anti-depressant effect was found in 6 out of 8 participants (Bewernick et al., 2017).

^e^In the complete sample of 5 patients, 3 were identical with patients of the study published by Merkl et al. (2013).

^f^One patient withdrew from study participation.

Up to one third of patients with MDD do not show significant symptom reduction in standard treatment and therefore have to be considered as treatment-resistant (Rush et al., 2006). Given the high prevalence of treatment-resistant major depression (TR-MDD), DBS-treatment of MDD is only performed in relatively rare experimental instances, which are generally included into clinical studies. Patients thus necessarily are research participants at the same time, highlighting the importance of preoperative valid informed consent. In the above studies, all included patients had a long history of chronic depression neither responding to pharmacological treatment, nor to psychotherapy or electroconvulsive therapy. For instance, the average length of the current depressive episode of patients included in one of the largest studies amounts to 10.8 years (Bewernick et al., 2010). Experimental treatment with DBS, although not yet approved for depression by the US Food and Drug Administration (FDA), thus can be considered a *last hope* for many patients, if the widely dismissed ablative neurosurgery is not taken into consideration due to its irreversibility.

Patient selection for DBS is always preceded by an extensive multi-professional screening process. The most important inclusion criteria, apart from diagnosis and treatment resistance, are the length of the current episode and severity of symptoms, typically measured with the Hamilton Depression Rating Scale (HDRS) or the Montgomery Åsberg Depression rating Scale (MADRS). The main exclusion criteria are current or past psychotic disorders, abnormal Magnetic Resonance Imaging (MRI) of the brain and any comorbid psychiatric, neurological or medical condition that could interfere with patients’ safety or compliance during treatment (Mayberg et al., 2005; Lozano et al., 2008; Malone et al., 2009; Schlaepfer et al., 2013).

In the light of DBS target regions associated with higher efficacy, continuous refinement of DBS-technology and novel applications on the horizon (Deeb et al., 2016), increasing medical interest in DBS correlates with a growing sensitivity to ethical questions raised by the use of invasive neuromodulation on psychiatric diseases. Accordingly, a consensus guideline on ethical and scientific conduct for psychiatric surgery has recently been published by an interdisciplinary group of experts (Nuttin et al., 2014). One of the principal concerns is that DBS could interfere with the ability of patients to make autonomous decisions and execute their free will. Consequently, the goal of this article is to evaluate the impact DBS might have on the capacity to decide and act autonomously for patients with treatment-resistant MDD. Special attention will be paid to the possibly autonomy-undermining effects depression has in itself, thus also potentially endangering valid Informed Consent—a key prerequisite for the procedure. Our approach is based on the conviction that with respect to the large differences between effects and side-effects of DBS, depending on the target of stimulation and the condition treated, a proper ethical evaluation of DBS-treatment should aim to be performed separately for every single indication, the target region used and the specific characteristics of both.

## Are Patients with MDD Competent to Give Informed Consent to Invasive Treatment?

The autonomy of patients undergoing DBS-treatment is not exclusively a matter of postoperative care. Since autonomy is one of the key principles of medical ethics and modern medical practice is widely based on it, autonomous decisions are supposed to stand at the very beginning of every medical intervention. Informed Consent was originally implemented to ensure that no patient would be harmed by unethical experiments or treatment against his or her will. From a rather defensive approach mostly aiming to protect participants of medical experiments, Informed Consent subsequently evolved into the main instrument to safeguard autonomous choices for patients in all matters of personal healthcare (Beauchamp, 2004). As such it has to be respected in the ethical evaluation of DBS-treatment for MDD as well. Since it is obvious that no person capable of expressing preferences can undergo neurosurgery against his or her will, the most relevant ethical question at this stage is, whether patients with MDD can be considered as autonomous agents in the sense that they are effectively able to provide valid Informed Consent. This is especially critical since there are good reasons to consider impaired autonomy as a key feature of severe psychiatric illness (DeGrazia, 1994). Also, it seems worth noting that DBS is not yet approved as a treatment for MDD in the United States, nor in Europe, implying that DBS in this case can only be performed within the framework of clinical trials. In giving consent to being treated, potential patients would also have to authorize a *research procedure*. This would require a certain appreciation of the experimental nature of this intervention and its partially unclear risk-benefit-ratio, posing an additional challenge to both the patient’s understanding and the quality of disclosure provided by healthcare professionals.

A wide consensus can be found among ethicists that at least three criteria have to be fulfilled for valid Informed Consent (Schöne-Seifert, 2007). First of all, healthcare professionals have to provide patients with all the information required for decision-making, including disclosure appropriate for the patient’s level of comprehension. Second, patients have to be competent to fully understand the information provided and must be able to decide on this basis. Finally, their decision has to be voluntary and free from manipulative influence or coercion. Assuming that good medical practice is at least likely to fulfill condition one and three and it is not ethically controversial that DBS is to be performed in such a context, our considerations will primarily focus on whether depressed patients are indeed competent to consent. This will also help clarify in which way patients with MDD are able to act autonomously and in which they are not. New efforts to discuss Informed Consent in the broader perspective of patients’ vulnerability will not be discussed here as for the focus of this article. For a concise overview of this recent attention-gaining approach, we recommend Bell et al. (2014).

According to the seminal work of Beauchamp and Childress, competence can be defined by four essential criteria:

*“Patients or prospective subjects are competent to make a decision if they have the capacity to understand the material information, to make a judgment about this information in light of their values, to intend a certain outcome, and to freely communicate their wishes to caregivers or investigators” (Beauchamp and Childress, 2009, p. 113)*.

Inability to give Informed Consent because of mental disorders is a very common problem in psychiatric practice and ethics (van Staden and Krüger, 2003). Several tests have been developed to assess patients’ competence, among them such well-established instruments as the MacArthur Competence Assessment Tool for Treatment (MacCAT-T; Grisso et al., 1997; Dunn et al., 2006). All in all, in psychiatric literature there has been little doubt about depressive patients’ competence to consent. In a line of thought reaching back to Jean-Étienne Esquirol, one of the fathers of modern psychiatry in the early 19th century, depression is perceived as a disorder which characteristically affects *the mood* and not the mind (Ehrenberg, 2009). Consequently, it is deemed unlikely to impair cognitive features like understanding material information or the ability to communicate freely in a way relevant to patients’ competence (Elliot, 2006). In fact, clinical experience shows that MDD might be associated with a slight decline in overall cognitive performance due to lack of concentration and general tiredness. However, only in the case of psychotic features, is it common to classify depressive patients as incompetent. Empirical research points in the same direction. Studies focusing on understanding and reasoning showed impairment in only 5.4% (understanding) respectively 7.6% (reasoning) of depressive inpatients consenting to treatment (Grisso and Appelbaum, 1995). Depressive inpatients asked to volunteer for research reached relatively high scores in the MacArthur Competence Assessment-Tool for clinical research and were found to be able to distinguish the levels of risk between studies (Cohen et al., 2004). In a similar vein, two studies on Informed Consent for ECT could not find any correlation between depression severity and decision-making capacity measured with the MacCAT-T (Lapid et al., 2003, 2004). Furthermore, it can be assumed that patients with MDD would also benefit from strategies to improve understanding and thus enhance the Informed Consent process in general. As a metaanalysis points out, research participants’ understanding of the information disclosed in the Informed consent process can be improved by either the use of multimedia or, most effectively, additional person-to-person contact between participants and healthcare professionals (Flory and Emanuel, 2004).

Hence, ethical concerns are raised mainly over the non-cognitive dimension of decision-making. For instance, it has been argued that MDD could significantly bias the evaluation of possible treatment results towards neglecting the likelihood of a positive outcome while overrating negative outcomes and treatment risks (Rudnick, 2002). In this case, one could assume patients’ judgment to depend on their overall negative perspective caused by depression rather than their true values. However, this argument overemphasizes the role of rational thinking and consciousness in decision-making. Most individuals do not decide exclusively by rationally weighing up pure facts. For the acceptance of a certain treatment, it can be crucial whether a physician seems trustworthy or if it just “feels right” to do it. Even if anxiety, a generally pessimistic perspective and maybe even desperation might have some influence on decision-making in the case of MDD, it seems questionable why this should be especially problematic in this context, while decisions in “normal” daily contexts led by the same irrational motives are considered adequate. Furthermore, anxiety and desperation are not at all specific traits of depressive patients, but in fact—understandably—a common feature of severe illness in general (Dunn et al., 2006). Thus, excluding patients from treatment or research *primarily* because of their desperation or anxiety could lead to the contradiction, that people who are the most in need of ultima-ratio interventions are the least likely to receive them. Apart from that, recent studies on depressive patients considering enrollment into DBS-treatment programs indicate that they rather tend to overrate their personal benefits and underestimate the likelihood of risks than vice versa (Leykin et al., 2011; Fisher et al., 2012). This widespread bias, known among ethicist and clinicians as *therapeutic misconception*, arises from individuals’ difficulties to distinguish between regular clinical care and research procedures with unclear therapeutic benefits (Appelbaum et al., 1982). Interestingly, depressive patients did not score worse than average non-psychiatric patients. In a small sample, severity of depressive symptoms even seemed to correlate with a more precise evaluation of risks and benefits (Fisher et al., 2012).

In a similar vein, it has been argued that MDD itself can change patients’ values, preferences and goals in a way that their decisions are not authentic (Rudnick, 2002; Elliot, 2006). There are several reasons to be skeptical of this argument. A general problem concerning authenticity as a criterion for the competence to consent is that it is only applicable ex-post, which means that patients’ competence would be verified after they have already decided. Assuming that this judgment would mainly depend on the content of the decision made, it is probable that physicians might tend to disrespect non-conforming choices by judging them to be inauthentic. For this reason, many ethicists argue that authenticity as a criterion for autonomous decisions runs the risk of promoting paternalism (Schöne-Seifert, 2007). Furthermore, severe illness as an extreme experience clearly has the potential to change someone’s goals or preferences in a relatively short amount of time. Authenticity, thus, cannot mean to expect patients to stick to their old opinions while their whole life is turned upside-down. Given that it is very natural to change one’s perspective on life in reaction to extreme situations, authenticity as a criterion becomes rather useless as a safeguard for competence. It seems neither theoretically plausible nor practically feasible to separate legitimate changes of mind from inauthentic shifts of preferences in the light of severe illness (Bielby, 2008). However, there is a third argument questioning MDD patients’ competence to consent, which seems to be more appropriate than the preceding ones. One main symptom of MDD is a general loss of interest in living, culminating in the worst case in suicide attempts or completed suicide. An often-cited example of how a weakened will to live can influence decision-making in medical matters is presented by Roth et al. (1977). The authors report a 49-year old woman who was asked to consent to electroconvulsive therapy because of MDD. When told that this treatment carries a risk of 1–3000 to die from complications, she articulated a surprising motivation to be treated by replying that she hoped to be the one (Roth et al., 1977). Whereas patients refusing treatment out of fear or by overrating negative outcomes at least show some concern for themselves, the patient in this extreme case displays an alarming lack of this fundamental interest in her own wellbeing. Choosing a treatment just because of the chance to die from adverse effects thwarts the very idea of Informed Consent. A patient using an instrument established to prevent harm *in order to get harmed* is not just executing his right of autonomous choice in a very uncommon way. He is rather refusing to act autonomously at all and therefore has to be considered incompetent to consent.

As a result, no argument seems strong enough to exclude patients with treatment-resistant MDD *collectively* from giving Informed Consent to DBS-treatment. Also, empirical data supports that autonomy in its sense of capacity to consent commonly seems not to be significantly impaired by MDD itself. We should take into consideration that expecting depressive patients to fulfill higher standards than mentally healthy patients would not only establish unfair access to regular or experimental treatment, but also reinforce stigmatization of this already disadvantaged group (Bell et al., 2014). The well-meant wish for special protection, when not reflected adequately, can easily relapse into old-fashioned (medical) paternalism.

On the other hand, regarding the invasiveness of DBS, the necessarily experimental character of treatment and as a concession to the current limitedness of empirical data, MDD-patients’ competence to give valid consent cannot simply be taken for granted. Individual evaluation of cognitive function, as should be part of every surgical or experimental treatment of psychiatric illness, is recommended in this context as well as extensive psychiatric evaluation, neuropsychological testing and multi-professional assessment including a thorough look at possible therapeutic misconception. Additionally, as shown in the example by Roth et al. (1977) it has to be ensured specifically that depressive patients’ decisions are motivated by the fundamental interest in their own wellbeing, which is the very essence of Informed Consent.

## Is DBS *per se* an Autonomy-Subverting Treatment?

**Figure 1:**
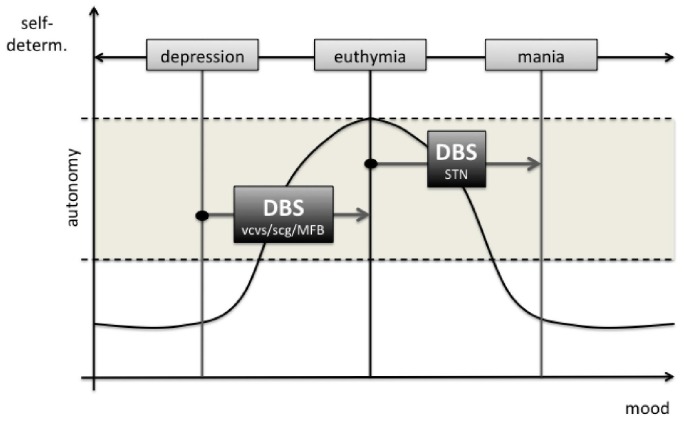
The potential effects of deep brain stimulation (DBS) of the different target regions on patients’ autonomy. In STN DBS mania is a rare (*ca*. 4%) side effect. Legend: vcvs, ventral capsule ventral striatum; scg, subgenual cingulate gyrus; MFB, medial forebrain bundle; STN, subthalamic nucleus.

As the ongoing popularity of literature and movies connected to the topic shows, the very idea of technical devices implanted in the human brain seems to cause discomfort to a considerable amount of people. Although general concerns towards DBS are by no means comparable to those fears famously expressed in novels like “The Manchurian Candidate” or “The Terminal Man”, similar questions are touched upon. Far from fictional scenarios of technically driven mind- and behavior-control, DBS also has to deal with the preoccupation that it might affect patients’ behavior in a way that their actions would no longer count as self-governed (Klaming and Haselager, 2013; Grant et al., 2014; Unterrainer and Oduncu, 2015). The goal of the following passage is to evaluate whether evidence can be found that DBS *for therapeutical use* could be a threat to patients’ autonomy. Due to a lack of empirical studies dedicated explicitly to this topic, our main focus will lie on a philosophically informed critical evaluation of cases of altered behavior during DBS-treatment. Although some cases at first sight suggest that DBS might influence decisional capacity, we hypothesize that it is in fact more likely for patients with MDD to benefit from DBS-Treatment with respect to their autonomy, given that MDD itself is a highly autonomy-subverting condition (see, Figure 1). A similar claim has recently been made for the treatment of obsessive-compulsive disorder, in which the overall positive effects of neuromodulation also seem to increase autonomy (De Ridder et al., 2016).

### Philosophical Background: Harry G. Frankfurt’s Hierarchical Model of Free Will

Going back to ancient philosophy and as a key concept of philosophical enlightenment, autonomy is a traditional issue of philosophy, recently discussed in the debate on free will and neuronal determinism. Among a variety of largely overlapping positions, the most prominent modern attempt to specify the characteristics of autonomous actions has been developed by US-philosopher Harry G. Frankfurt (Frankfurt, 1988). Frankfurt’s *hierarchical model of free will* belongs to the so called *internalist* wing of the debate. According to internalists’ point of view, the key feature of autonomous agency is that the agent’s motives leading to particular actions somehow cohere with a framework of more general higher motives and attitudes. The latter can be called *internal* in the sense that they are mental states belonging to the agent, which are closely bound to his or her personality (Buss, 2014)^2^. They have been referred to as *higher-order desires* (Frankfurt, 1988), evaluational judgments (Watson, 1975), long term plans (Bratman, 2007) or character traits (Dworkin, 1988). Following this train of thought, a person who acts in accordance with her own—however labeled—higher-order attitudes, ultimately acts in accordance with herself, thus literally being *autonomous* in the original ancient Greek translation of *self-governing*. While internalists focus on the two-level structure of basic desires or actions and corresponding higher order desires or attitudes, taking *coherence* between the two levels as the benchmark of autonomy, *externalist* approaches, representing the other prominent wing of the debate, place much more emphasis on rationality of the agents and their higher motives themselves. According to their shared intuition, agents can only be autonomous, if they are conscious of and able to articulate logical, sound reasons for their doing (Fischer and Ravizza, 1993; Nelkin, 2007) or are at least potentially able to reason about it in an appropriate way (Christman, 1991, 1993; Mele, 1993). Consequently, for these authors autonomous action is not primarily a matter of personal preferences, but most importantly has to fulfill certain *external* criteria: to match the objective world and to follow the laws of logic.

There are several reasons to favor internalist approaches like Frankfurts’ hierarchical model of free will. Generally speaking, externalists’ main interest lies in identifying the conditions under which an agent can be held responsible for his or her actions, while neglecting engagement with the process of autonomous decision and action itself. As it is necessary to have at least a minimal theoretical understanding of how autonomous actions really take place in order to evaluate possible autonomy-subverting effects of DBS, it is evident why externalist approaches are of little use for our purpose.

Frankfurt’s concept of autonomy is not only the most established internalist approach, it also best matches the requirements of our endeavor and the kind of data we have to deal with. Frankfurt suggests a clear and comprehensible model of autonomous agency, which is remarkably close to the everyday-experience of self-governed agency. It allows a clear focus on agents’ behavior and their attitudes towards it. This is especially important given that we have no other option than drawing inferences from literature without knowing the individuals involved personally, thus being unable to verify the logical soundness of their motives or their ability to reason. Since there are no specific studies on DBS and autonomy, our aim is to take a close look at harmful or otherwise abnormal behavior which occurred under DBS-treatment and then question it for signs of the agents’ underlying higher-order attitudes.

According to Frankfurt, persons can be distinguished from other living beings who are not persons by being capable of having two different classes of desires and to reflect on them. Whereas first-order desires have the structure “A wants to X” with X representing a certain action, second-order desires refer back to first-order-desires a person does or does not want to have. A person is acting autonomously when her first-order desires expressed in effective action are fitting to the framework of her second-order desires. The key feature of free will, therefore, is that a person *identifies* with her actions, meaning that she truly wants to do what she effectively does and, on the other hand, really does not want to do what she passes by. According to Frankfurt, an action is hence autonomous, only if it coheres to the acting person’s preferences, values and goals as represented by her second-order desires.

### Autonomy Under DBS: A Critical Review of Empirical Data

Due to a lack of systematic empirical research on autonomy-subverting effects of any kind of neuromodulation, evaluating the risks of DBS-treatment to patients’ autonomy in the case of MDD naturally faces a major difficulty: the only available data containing information about behavior under DBS indicating impaired autonomy originate from patients receiving treatment for different conditions. The predictive power of any evaluation thus relies on careful selection of cases which are at least in some respect comparable. The majority of studies and case reports dealing with troubled decision-making and abnormal behavior under the influence of DBS are derived from treatment of Parkinson-Disease (PD). The most relevant subgroup of patients for our purpose are those receiving DBS of the Subthalamic Nucleus (STN), which is embedded in similar neuronal circuits as DBS for MDD aims to modulate. Among several known side effects of STN-stimulation, the second most frequent psychiatric side effect (after depression) is hypomania with an estimated rate of 4% (Temel et al., 2006). According to ICD-10, core symptoms of hypomania are abnormally increased energy and activity under persistent elevation of mood, becoming manifest in behavioral changes such as heightened sociability, talkativeness, overfamiliarity, increased sexual energy or decreased need for sleep. Correspondingly, Mandat et al. (2006) report two cases of hypomania under STN-DBS leading to detrimental behavior of two male PD-patients. Seventy-two-year-old patient 1 purchased a new car, ignoring that he would never be able to drive it as he had been physically disabled for years. He also arranged to be visited by a prostitute, clearly disregarding the rules of his nursing home. Forty-five-year old patient 2, lacking any history of psychiatric disorders or criminal behavior, broke into a parked car in the middle of a crowded street. Similarly, Romito et al. (2002) report two male PD-patients with STN-DBS displaying behavioral changes associated with general symptoms of mania. Whereas patient 2 showed a wide range of abnormal sexual behavior, most remarkably inappropriate seductive behavior toward female staff, patient 1 started writing religious poems despite never having shown any interest in religion. He also began to purchase items he did not need, to plan hazardous business investments and to drive his car in a reckless manner (Romito et al., 2002, p. 1372).

Although it seems evident that the patients in these cases are not acting autonomously while in a hypomanic state, the application of Frankfurt’s model to such reports is quite difficult. According to Frankfurt, the most important criterion for autonomy is coherence of the performed actions with the second-order desires of the agent. Knowing the patients only by case report, we have no access to the framework of their second-order desires. For instance, it is hard to tell whether Romito’s patient 2, when writing religious poems, is just driven by some spontaneous manic fantasy or if he might be giving in to suppressed wishes. Nevertheless, we can assume that at least some of the abnormal actions displayed contradicted the higher-order desires of the agents. Wasting money on unneeded items, risking to die in a car crash or being charged with sexual harassment are very unlikely to correspond with the preferences of virtually anyone. Given that this behavior did not occur before, it appears that patients acting this way thus apparently have serious problems to act in accordance with their higher-order desires, following random impulses instead. Mandat’s patient 2, who broke into a parked car, was later found unable to explain why he did it. Taking this lack of adequate reasons as a sign for a lack of second-order desires in favor of breaking into cars, the best explanation for this patient’s behavior is that he was unable to resist a sudden impulse. According to Frankfurt, it is the key feature of personal autonomy to evaluate first-order desires in the light of second-order desires, which usually leads to actions compatible with a person’s higher-order desires. In the cases mentioned, this mechanism seems to be impaired by impulses bypassing rational assessment. Patients tending to give in to impulses triggered by external factors (e.g., an empty highway, attractive medical staff, opportunities for spontaneous purchases) without evaluating them, thus carry a high risk for non-autonomous actions.

Having identified decreased impulse control as a possible autonomy-subverting adverse-effect of DBS, further evaluation needs to assess, if there are also examples of impaired autonomous decision-making during DBS-treatment other than manic or hypomanic states. Indeed, several cases of abnormal behavior under DBS have been reported which evidently were associated with impaired impulse control. Sensi et al. (2004) describe a patient showing explosive-aggressive behavior undergoing STN-DBS, which they explicitly relate to disturbed impulse control. On the second day postoperative, the 64-year old male exhibited spontaneous aggressive outbursts including physical attacks towards medical staff and his own family. Furthermore, he displayed kleptomaniac behavior trying to steal electric wires and bath towels. After they had found out that his aggressive behavior correlated with the strength of neurostimulation, the treating physicians eventually gained control of psychiatric symptoms through a moderate dose of antipsychotic medication (Quetiapine 100 mg/d). Supporting Sensi et al.’s (2004) interpretation, both ICD-10 and DSM-IV rank kleptomania and intermittent explosive disorder among impulse control disorders. Another kind of abnormal behavior belonging to this category is pathological gambling, which has also been found in PD-Patients undergoing DBS-treatment. According to Frankfurt, who uses the figure of the *unwilling addict* as the prime example of deficient autonomy (Frankfurt, 1988), it is highly plausible to consider pathological gambling an almost paradigmatic example of non-autonomous behavior. Typically, pathological gamblers not only act *against* their assumed second-order desires. In a certain sense one could argue that they rather act *at the expense of their second-order desires as such*, successively destroying their financial well-being and putting at stake all kinds of interpersonal relationships. In this regard, Smeding et al. (2007) report a 63-year old male patient who developed pathological gambling under the impact of STN-Stimulation. According to his family, the patient previously was “as stingy as a Dutchman”. Within 1 month of his treatment, he started to gamble away considerable amounts of money, which resulted in increasing debts, the sale of his house and his wife wanting divorce. His desperate situation finally culminated in three suicide attempts, prompting his admission to the neurological ward, where his urge to gamble ceased after modification of his Parkinson medication. Apart from these rather extreme cases of impaired impulse control, STN-DBS might also impair decision-making in a less obvious way. A study based on neuropsychological tests suggests that STN-DBS can interfere with the patients’ ability to stop and think when confronted with difficult decisions, thus leading to suboptimal choices (Frank et al., 2007). However, given that patients tended towards impulsive decisions especially in win-win-situations, in which only slight extra-benefits could be gained by careful selection, it seems questionable if this study actually indicates a severe threat to autonomy in any relevant sense.

Although there seems to be a wide range of autonomy-subverting side effects associated with DBS-treatment at first sight, the role of DBS in all these cases remains controversial. At least two more factors need to be taken into account for a proper evaluation. First, every patient mentioned was suffering from advanced PD and thus from a condition which severely affects dopaminergic transmission in the brain. PD itself can—at a certain disease stage—have exactly the same symptoms. Second, every patient had a history of dopamine replacement therapy and in the majority of cases medication was still being used in addition to DBS. The combination of these two factors was identified as a potential risk for impulse control disorders years ago (Voon et al., 2011). Dopaminergic dysregulation syndrome, resulting both from neurodegenerative effects of PD and longtime dopamine replacement therapy, has been discussed as a possible explanation for behavioral changes and reduced impulse control emerging independently of DBS-treatment (O’Sullivan et al., 2009; Katzenschlager, 2011). Correspondingly, Smeding et al. (2007) patient’s pathological gambling eventually resolved after pergolide treatment was stopped, while DBS was continued. However, there have even been several cases in which impulse control disorders improved under DBS (Ardouin et al., 2006). All in all it is hence plausible to regard STN-DBS as just one of several factors, which, in combination, can possibly cause impairment of impulse control in *some* individuals. The same applies to manic or hypomanic states, which also occurred mainly among patients who had already been suffering from conditions affecting the limbic dopamine system. Also taking into account that the vast majority of patients undergoing DBS did not show any kind of autonomy subverting complications, it thus seems appropriate not to generally criticize DBS as a threat to autonomy. Taking seriously the examples of impaired decision-making rather should result in raising overall awareness for this category of adverse effects, including them into Informed Consent procedures and advancing standards of postoperative care.

Whereas it is questionable which role DBS has played in the mentioned cases and to which extent results originating from STN-DBS on PD-patients can be applied to DBS of patients with MDD, the few pilot studies already indicated widespread positive effects of DBS on the autonomy of depressive patients. According to Frankfurt’s concept, patients with MDD can be considered non-autonomous in a very distinct sense: although depression does not affect most patients’ capability to make rational decisions, reflected in depressive patients’ ability to give valid Informed Consent, it is a general feature of MDD that patients are unable to act as they truly want to. This has to do with its main symptoms, which are lowering of mood, loss of interest due to general anhedonia and reduction of energy with a decrease in activity. A typical patient therefore might actually wholeheartedly wish to leave his bed, go to work or meet with friends etc., but still none or just very few of these desires will lead to goal directed action. For this reason, patients with MDD can be conceptualized as persons with an intact framework of second-order desires, who have a significantly reduced ability to convert them into effective first-order desires and thus to act autonomously. This very impairment often entails loss of work, financial problems, damaged partnerships and social isolation. Given that there is a negative correlation between depression and autonomy, response or remission of patients undergoing DBS-treatment can be regarded as strong indicators for an increase in autonomy as well. In fact, about half of the patients included in the main pilots responded to treatment with a decrease of at least 50% on HDRS. Even non-responders have been reported to show signs of improving goal directed action (e.g., increase of activity, making new acquaintances, resuming part-time work; Bewernick et al., 2010). Thus, in the case of patients with MDD, DBS is more likely to partially restore autonomy than to subvert it, re-enabling patients to put their desires into action and leading a life which corresponds at least more closely to their own preferences than years of depressive stagnation presumably did.

## Conclusion: Autonomy as Gradual

As a result of our considerations, we propose to regard the majority of patients with MDD as likely to be capable of autonomous decision-making but very unlikely to be fully able to effectively act according to their own will, whenever acting includes significant personal effort. Taking into account that MDD is a highly autonomy-subverting condition, DBS thus rather seems to be a chance to restore some sovereignty in everyday life than a threat to autonomy. Though there have been some cases of partially impaired autonomy among PD-patients, potential risks of DBS in this respect seem to be overcome by anticipated benefits indicated by the pilot studies of MDD-treatment.

From a strictly clinical point of view, it is worth noting that significant improvements in daily and social life (e.g., increase of activity, establishing a daily structure, reengagement in gainful employment), which can be seen as major contributions to an overall increase of autonomy, might not be displayed adequately in standard outcome measurements. Improvements of this kind are poorly reflected in commonly used symptom-based rating scales like HRDS or MADRS. These instruments have been designed originally to monitor the effects of pharmacotherapy on “everyday” depressive patients, but not for the extreme case of treatment-resistant MDD (Bewernick et al., 2010). Exclusively measuring the effects of DBS with symptom-based scales thus could result in a paradoxical situation: patients who subjectively experience benefits of high personal relevance might be nonetheless considered objective non-responders (Bewernick et al., 2017). In a similar vein, minor but relevant improvements might be noticed best by close relatives and consequently also not be reflected in outcome measurement (Crowell et al., 2015). In line with the recently published consensus guideline for psychiatric surgery, this highlights the importance of quality-of-life measurement for the general outcome assessment of DBS for MDD (Nuttin et al., 2014). Furthermore, these findings support the claim to include individually defined treatment goals—which can of course be diverse and continually evolving—in the evaluation of overall effects of DBS-treatment (Kubu and Ford, 2012). A shift towards the individualization of outcome assessment by using more sensitive tools for improvements in daily living as well as personally defined treatment goals would facilitate the proper assessment of a possible increase of autonomy due to DBS-treatment too. Autonomy in its broadest and maybe most relevant sense means the capacity to live a self-governed life which accords as much as possible with the preferences of the agent, making it a life, which is subjectively worth living. Therefore, undergoing DBS-treatment in order to increase quality of life and to reach certain self-defined goals would not only overlap with the idea of autonomy but can in itself already be seen as a first step of regaining autonomy.

From a philosophical point of view, the example of severely depressive patients being able to decide autonomously while heavily impaired in their performance of autonomous actions, underlines that autonomy should be regarded as *gradual*. Autonomy cannot be appropriately conceptualized as an ability which is either completely lacking or fully intact. Both extremes are located at the very ends of a broad continuum. Persons reaching one of these extremes presumably are rare exceptions, given that normal, healthy agents also regularly perform actions which contradict their second-order desires. For our case, insisting on autonomy as gradual has two important implications. First, it would be more accurate to discuss DBS and its wanted or unwanted effects in terms such as “*reducing”* or *“increasing”* than “threatening”, “losing” or “restoring” autonomy, which all implicitly refer to autonomy as a whole. Second, acknowledging the striking deficits in autonomy caused by MDD, the main therapeutic goal of DBS relative to patients’ autonomy can only lie in *maximizing* their ability to lead a life according to their own will (Beeker, 2014). Assuming that severe motor impairments due to PD undermine self-determined living in a similar way as MDD does, the treating physicians in the cases analyzed therefore did right to continue stimulation despite the observed side effects. Having achieved significant motor benefits, they carefully adjusted stimulation parameters, optimized additional medication or just waited for adaption by way of neuronal plasticity, instead of immediately ending DBS. Remarkably, in all cases a lasting gain of overall autonomy finally achieved through successful treatment of motor symptoms thus was preceded by an episode of partially diminished autonomy. The same could apply to DBS-treatment of MDD. An evaluation of the outcome of patients always has to take into consideration that MDD itself—and therefore the condition every treatment result has to be compared to—is a condition which impedes patients living autonomously in any meaningful sense. For treating physicians, maximizing patients’ autonomy in this context would mean primarily to aim for remission of depressive symptoms while carefully managing possible adverse effects. If patients are clinically benefiting from stimulation, effects which might reduce autonomy to some degree should be tolerated as long as there is reasonable hope of eventually reaching overall and long-term gains of autonomy. Even moderate persistent side effects could be tolerated, if in accordance with the will of a patient or if negligible from a broader quality-of-life perspective on autonomy. A slight overall increase in impulsiveness or a tendency towards suboptimal choices in win-win-situations might look like small disturbances to most patients, if in exchange chronic symptoms remit and theses patients are enabled to lead a relatively normal life again.

## Author Contributions

TB drafted the manuscript, TES and VAC substantially contributed to the conception of the work as well as its critical revision for important intellectual content. All authors approve the final version to be published and agree to be accountable for all aspects of the work, its accuracy and integrity.

## Conflict of Interest Statement

TB has no conflict of interest. TES was supported in the last five years in part by a grant from Medtronic, Inc., a manufacturer of DBS devices and the Hope for Depression Research Foundation and the Institute for Affective Neuroscience. VAC has occasionally received travel support and honoraria for lecturing and consulting from Medtronic (USA, Europe) and Boston Scientific (USA). VAC has ongoing IIT’s with Medtronic and Boston Scientific unrelated to this work.

## Footnotes

^1^For a current review see Schlaepfer et al. (2014).

^2^http://plato.stanford.edu/archives/win2014/entries/personal-autonomy/ (accessed October 12, 2016).

## References

[B1] Accolla E. A. Aust S. Merkl A. Schneider G.-H. Kühn A. A. Bajbouj M.. (2016). Deep brain stimulation of the posterior gyrus rectus region for treatment resistant depression. J. Affect. Disord. 194, 33–37. 10.1016/j.jad.2016.01.022 26802505

[B2] Appelbaum P. S. Roth L. H. Lidz C. (1982). The therapeutic misconception: informed consent in psychiatric research. Int. J. Law Psychiatry 5, 319–329. 10.1016/0160-2527(82)90026-7 6135666

[B3] Ardouin C. Voon V. Worbe Y. Abouazar N. Czernecki V. Hosseini H.. (2006). Pathological gambling in Parkinson’s disease improves on chronic subthalamic nucleus stimulation. Mov. Disord. 21, 1941–1946. 10.1002/mds.21098 16972268

[B5] Beauchamp T. L. (2004). “History of informed consent,” in Encyclopedia of Bioethics, ed. Post S. G. (New York, NY: Macmillan Publishers), 1271–1277.

[B4] Beauchamp T. L. Childress J. F. (2009). Principles of Biomedical Ethics. 6th Edn. New York, NY: Oxford University Press

[B6] Beeker T. (2014). Tiefe Hirnstimulation als Ultima Ratio? Eine Medizinethische Untersuchung am Beispiel der Therapieresistenten Depression. Münster: Mentis.

[B7] Bell E. Racine E. Chiasson P. Dufourcq-Brana M. Dunn L. B. Fins J. J.. (2014). Beyond consent in research. Revisiting vulnerability in deep brain stimulation for psychiatric disorders. Camb. Q. Healthc. Ethics 23, 361–368. 10.1017/S0963180113000984 24865371

[B8] Bergfeld I. O. Mantione M. Hoogendoorn M. L. Ruhé H. G. Notten P. van Laarhoven J.. (2016). Deep brain stimulation of the ventral anterior limb of the internal capsule for treatment-resistant depression: a randomized clinical trial. JAMA Psychiatry 73, 456–464. 10.1001/jamapsychiatry.2016.0152 27049915

[B9] Bewernick B. H. Hurlemann R. Matusch A. Kayser S. Grubert C. Hadrysiewicz B.. (2010). Nucleus accumbens deep brain stimulation decreases ratings of depression and anxiety in treatment-resistant depression. Biol. Psychiatry 67, 110–116. 10.1016/j.biopsych.2009.09.013 19914605

[B10] Bewernick B. H. Kayser S. Gippert S. M. Switala C. Coenen V. A. Schlaepfer T. E. (2017). Deep brain stimulation to the medial forebrain bundle for depression- long-term outcomes and a novel data analysis strategy. Brain Stimul. 10, 664–671. 10.1016/j.brs.2017.01.581 28259544

[B11] Bielby P. (2008). Competence and Vulnerability in Biomedical Research. Dordrecht, AN: Springer.

[B12] Bratman M. E. (2007). Structures of Agency: Essays. Oxford: Oxford University Press.

[B13] Buss S. (2014). “Personal autonomy,” in The Stanford Encyclopedia of Philosophy. Available online at: http://plato.stanford.edu/archives/win2014/entries/personal-autonomy/

[B14] Christman J. (1991). Autonomy and personal history. Can. J. Philos. 21, 1–24. 10.1080/00455091.1991.10717234

[B15] Christman J. (1993). Defending historical autonomy: a reply to professor Mele. Can. J. Philos. 23, 281–290. 10.1080/00455091.1993.10717321

[B16] Coenen V. A. Schlaepfer T. E. Maedler B. Panksepp J. (2011). Cross-species affective functions of the medial forebrain bundle-implications for the treatment of affective pain and depression in humans. Neurosci. Biobehav. Rev. 35, 1971–1981. 10.1016/j.neubiorev.2010.12.009 21184778

[B17] Cohen B. J. McGarvey E. L. Pinkerton R. C. Kryzhanivska L. (2004). Willingness and competence of depressed and schizophrenic inpatients to consent to research. J. Am. Acad. Psychiatry Law 32, 134–143. 15281414

[B18] Crowell A. L. Garlow S. J. Riva-Posse P. Mayberg H. S. (2015). Characterizing the therapeutic response to deep brain stimulation for treatment-resistant depression: a single center long-term perspective. Front. Integr. Neurosci. 9:41. 10.3389/fnint.2015.00041 26124710 PMC4466607

[B19] De Ridder D. Vanneste S. Gillett G. Manning P. Glue P. Langguth B. (2016). Psychosurgery reduces uncertainty and increases free will? A review. Neuromodulation 19, 239–248. 10.1111/ner.12405 26899938

[B20] Deeb W. Giordano J. J. Rossi P. J. Mogilner A. Y. Gunduz A. Judy J. W.. (2016). Proceedings of the fourth annual deep brain stimulation think tank: a review of emerging issues and technologies. Front. Integr. Neurosci. 10:38. 10.3389/fnint.2016.00038 27920671 PMC5119052

[B21] DeGrazia D. (1994). Autonomous action and autonomy-subverting psychiatric conditions. J. Med. Philos. 19, 279–297. 10.1093/jmp/19.3.279 7964212

[B22] Dougherty D. D. Rezai A. R. Carpenter L. L. Howland R. H. Bhati M. T. O’Reardon J. P.. (2015). A randomized sham-controlled trial of deep brain stimulation of the ventral capsule/ventral striatum for chronic treatment-resistant depression. Biol. Psychiatry 78, 240–248. 10.1016/j.biopsych.2014.11.023 25726497

[B23] Dunn L. B. Nowrangi M. A. Palmer B. W. Jeste D. V. Saks E. R. (2006). Assessing decisional capacity for clinical research or treatment: a review of instruments. Am. J. Psychiatry 163, 1323–1334. 10.1176/appi.ajp.163.8.1323 16877642

[B24] Dworkin G. (1988). The Theory and Practice of Autonomy. New York, NY: Cambridge University Press.

[B25] Ehrenberg A. (2009). The Weariness of the Self: Diagnosing the History of Depression in the Contemporary Age. Montreal: McGill-Queen’s University Press.

[B26] Elliot C. (2006). “Caring about risks: are severely depressed patients competent to consent to research?,” in An Anthology of Psychiatric, eds Green S. A. Bloch S. (Oxford: Oxford University Press), 463–465.

[B27] Fenoy A. J. Schulz P. Selvaraj S. Burrows C. Spiker D. Cao B.. (2016). Deep brain stimulation of the medial forebrain bundle: distinctive responses in resistant depression. J. Affect. Disord. 203, 143–151. 10.1016/j.jad.2016.05.064 27288959

[B28] Fischer J. Ravizza M. (1993). Perspectives on Moral Responsibility. Ithaca: Cornell University Press.

[B29] Fisher C. E. Dunn L. B. Christopher P. P. Holtzheimer P. E. Leykin Y. Mayberg H. S.. (2012). The ethics of research on deep brain stimulation for depression: decisional capacity and therapeutic misconception. Ann. N Y Acad. Sci. 1265, 69–79. 10.1111/j.1749-6632.2012.06596.x 22812719 PMC3624886

[B30] Flory J. Emanuel E. (2004). Interventions to improve research participants’ understanding in informed consent for research: a systematic review. JAMA 292, 1593–1601. 10.1001/jama.292.13.1593 15467062

[B31] Frank M. J. Samanta J. Moustafa A. Sherman S. J. (2007). Hold your horses: impulsivity, deep brain stimulation and medication in parkinsonism. Science 318, 1309–1312. 10.1126/science.1146157 17962524

[B32] Frankfurt H. G. (1988). “Freedom of the will and the concept of a person,” in What is a Person? ed. Goodman M. F. (Clifton, NJ: Humana Press), 127–144.

[B33] Gildenberg P. L. (2002). Spiegel and wycis—the early years. Stereotact. Funct. Neurosurg. 77, 11–16. 10.1159/000064587 12378049

[B34] Gildenberg P. L. Krauss J. K. (2009). “History of stereotactic surgery,” in Textbook of Stereotactic and Functional Neurosurgery, eds Lozano A. M. Gildenberg P. L. Tasker R. R. (Berlin: Springer), 1–44.

[B35] Grant R. A. Halpern C. H. Baltuch G. H. O’Reardon J. P. Caplan A. (2014). Ethical considerations in deep brain stimulation for psychiatric illness. J. Clin. Neurosci. 21, 1–5. 10.1016/j.jocn.2013.04.004 24055023

[B36] Grisso T. Appelbaum P. S. (1995). The MacArthur treatment competence study. III: abilities of patients to consent to psychiatric and medical treatments. Law Hum. Behav. 19, 149–174. 10.1007/bf01499323 11660292

[B37] Grisso T. Appelbaum P. S. Hill-Fotouhi C. (1997). The MacCAT-T: a clinical tool to assess patients’ capacities to make treatment decisions. Psychiat. Serv. 48, 1415–1419. 10.1176/ps.48.11.1415 9355168

[B38] Holtzheimer P. E. Kelley M. E. Gross R. E. Filkowski M. M. Garlow S. J. Barrocas A.. (2012). Subcallosal cingulate deep brain stimulation for treatment-resistant unipolar and bipolar depression. Arch. Gen. Psychiatry 69, 150–158. 10.1001/archgenpsychiatry.2011.1456 22213770 PMC4423545

[B39] Katzenschlager R. (2011). Dopaminergic dysregulation syndrome in Parkinson’s disease. J. Neurol. Sci. 310, 271–275. 10.1016/j.jns.2011.07.012 21868039

[B40] Klaming L. Haselager P. (2013). Did my brain implant make me do it? Questions raised by DBS regarding psychological continuity, responsibility for action and mental competence. Neuroethics 6, 527–539. 10.1007/s12152-010-9093-1 24273622 PMC3825573

[B41] Kubu C. S. Ford P. J. (2012). Beyond mere symptom relief in deep brain stimulation: an ethical obligation for multifaceted assesment of outcome. AJOB Neurosci. 3, 44–49. 10.1080/21507740.2011.633960 22737593 PMC3377486

[B43] Lapid M. I. Rummans T. A. Pankratz V. S. Appelbaum P. S. (2004). Decisional capacity of depressed elderly to consent to electroconvulsive therapy. J. Geriatr. Psychiatry Neurol. 17, 42–46. 10.1177/0891988703261996 15018698

[B42] Lapid M. I. Rummans T. A. Poole K. L. Pankratz V. S. Maurer M. S. Rasmussen K. G.. (2003). Decisional capacity of severely depressed patients requiring electroconvulsive therapy. J. ECT 19, 67–72. 10.1097/00124509-200306000-00002 12792453

[B44] Leykin Y. Christopher P. P. Holtzheimer P. E. Appelbaum P. S. Mayberg H. S. Lisanby S. H.. (2011). Participants perceptions of deep brain stimulation research for treatment-resistant depression: risks, benefits, and therapeutic misconception. AJOB Prim. Res. 2, 33–41. 10.1080/21507716.2011.627579 26225215 PMC4516276

[B45] Lozano A. M. Mayberg H. S. Giacobbe P. Hamani C. Craddock R. C. Kennedy S. H. (2008). Subcallosal cingulate gyrus deep brain stimulation for treatment-resistant depression. Biol. Psychiatry 64, 461–467. 10.1016/j.biopsych.2008.05.034 18639234

[B46] Malone D. A. Dougherty D. D. Rezai A. R. Carpenter L. L. Friehs G. M. Eskandar E. N.. (2009). Deep brain stimulation of the ventral capsule/ventral striatum for treatment-resistant depression. Biol. Psychiatry 65, 267–275. 10.1016/j.biopsych.2008.08.029 18842257 PMC3486635

[B47] Mandat T. S. Hurwitz T. Honey C. R. (2006). Hypomania as an adverse effect of subthalamic nucleus stimulation: report of two cases. Acta Neurochirurgica 148, 895–898. 10.1007/s00701-006-0795-4 16763733

[B48] Mayberg H. S. Lozano A. M. Voon V. McNeely H. E. Seminowicz D. Hamani C.. (2005). Deep brain stimulation for treatment-resistant depression. Neuron 45, 651–660. 10.1016/j.neuron.2005.02.014 15748841

[B49] Mele A. (1993). History and personal autonomy. Can. J. Philos. 23, 271–280. 10.1080/00455091.1993.10717320

[B50] Merkl A. Schneider G. H. Schönecker T. Aust S. Kühl K. P. Kupsch A.. (2013). Antidepressant effects after short-term and chronic stimulation of the subgenual cingulate gyrus in treatment-resistant depression. Exp. Neurol. 249, 160–168. 10.1016/j.expneurol.2013.08.017 24012926

[B51] Nelkin D. (2007). Do we have a coherent set of intuitions about moral responsibility? Midwest. Stud. Philos. 31, 243–259. 10.1111/j.1475-4975.2007.00159.x

[B52] Nuttin B. Wu H. Mayberg H. Hariz M. Gabriëls L. Galert T.. (2014). Consensus on guidelines for stereotactic neurosurgery for psychiatric disorders. J. Neurol. Neurosurg. Psychiatry 85, 1003–1008. 10.1136/jnnp-2013-306580 24444853 PMC4145431

[B53] O’Sullivan S. S. Evans A. H. Lees A. J. (2009). Dopamine dysregulation syndrome: an overview of its epidemiology, mechanisms and management. CNS Drugs 23, 157–170. 10.2165/00023210-200923020-00005 19173374

[B54] Panksepp J. (1998). Affective Neuroscience—The Foundation of Human and Animal Emotions. New York, NY: Oxford University Press.

[B55] Puigdemont D. Pérez-Egea R. Portella M. J. Molet J. de Diego-Adeliño J. Gironell A.. (2012). Deep brain stimulation of the subcallosal cingulate gyrus: further evidence in treatment-resistant major depression. Int. J. Neuropsychopharmacol. 15, 121–133. 10.1017/S1461145711001088 21777510

[B56] Ramasubbu R. Anderson S. Haffenden A. Chavda S. Kiss Z. H. (2013). Double-blind optimization of subcallosal cingulate deep brain stimulation for treatment-resistant depression: a pilot study. J. Psychiatry Neurosci. 38, 325–332. 10.1503/jpn.120160 23527884 PMC3756116

[B57] Romito L. M. Raja M. Daniele A. Contarino M. F. Bentivoglio A. R. Barbier A.. (2002). Transient mania with hypersexuality after surgery for high frequency stimulation of the subthalamic nucleus in Parkinson’s disease. Mov. Disord. 17, 1371–1374. 10.1002/mds.10265 12465087

[B58] Roth L. H. Meisel A. Lidz C. W. (1977). Tests of competency to consent to treatment. Am. J. Psychiatry 134, 279–284. 10.1176/ajp.134.3.279 842704

[B59] Rudnick A. (2002). Depression and competence to refuse psychiatric treatment. J. Med. Ethics 28, 151–155. 10.1136/jme.28.3.151 12042398 PMC1733573

[B60] Rush A. J. Trivedi M. H. Wisniewski S. R. Nierenberg A. A. Stewart J. W. Warden D.. (2006). Acute and longer-term outcomes in depressed outpatients requiring one or several treatment steps: a STAR*D report. J. Psychiatr. Res. 163, 1905–1917. 10.1176/appi.ajp.163.11.1905 17074942

[B62] Schlaepfer T. E. Bewernick B. H. Kayser S. Hurlemann R. Coenen V. A. (2014). Deep brain stimulation of the human reward system for major depression—rationale, outcomes and outlook. Neuropsychopharmacology 39, 1303–1314. 10.1038/npp.2014.28 24513970 PMC3988559

[B61] Schlaepfer T. E. Bewernick B. H. Kayser S. Mädler B. Coenen V. A. (2013). Rapid effects of deep brain stimulation for treatment-resistant major depression. Biol. Psychiatry 73, 1204–1212. 10.1016/j.biopsych.2013.01.034 23562618

[B63] Schlaepfer T. E. Cohen M. X. Frick C. Kosel M. Brodesser D. Axmacher N.. (2008). Deep brain stimulation to reward circuitry alleviates anhedonia in refractory major depression. Neuropsychopharmacology 33, 368–377. 10.1038/sj.npp.1301408 17429407

[B64] Schoene-Bake J.-C. Parpaley Y. Weber B. Panksepp J. Hurwitz T. A. Coenen V. A. (2010). Tractographic analysis of historical lesion surgery for depression. Neuropsychopharmacology 35, 2553–2563. 10.1038/npp.2010.132 20736994 PMC3055575

[B65] Schöne-Seifert B. (2007). Grundlagen der Medizinethik. Stuttgart: Kröner.

[B66] Sensi M. Eleopra R. Cavallo M. A. Sette E. Milani P. Quatrale R.. (2004). Explosive-aggressive behavior related to bilateral subthalamic stimulation. Parkinsonism Relat. Disord. 10, 247–251. 10.1016/j.parkreldis.2004.01.007 15120100

[B67] Smeding H. M. M. Goudriaan A. E. Foncke E. M. J. Schuurman P. R. Speelman J. D. Schmand B. (2007). Pathological gambling after bilateral subthalamic nucleus stimulation in Parkinson disease. J. Neurol. Neurosurg. Psychiatry 78, 517–519. 10.1136/jnnp.2006.102061 17210626 PMC2117849

[B68] Temel Y. Kessels A. Tan S. Topdag A. Boon P. Visser-Vandewalle V. (2006). Behavioural changes after bilateral subthalamic stimulation in advanced Parkinson disease: a systematic review. Parkinsonism Relat. Disord. 12, 265–272. 10.1016/j.parkreldis.2006.01.004 16621661

[B69] Unterrainer M. Oduncu F. S. (2015). The ethics of deep brain stimulation (DBS). Med. Health. Care Philos. 18, 475–485. 10.1007/s11019-015-9622-0 25597042

[B70] van Staden C. W. Krüger C. (2003). Incapacity to give informed consent owing to mental disorder. J. Med. Ethics 29, 41–43. 10.1136/jme.29.1.41 12569195 PMC1733664

[B71] Voon V. Mehta A. R. Hallett M. (2011). Impulse control disorders in Parkinson’s disease: recent advances. Curr. Opin. Neurol. 24, 324–330. 10.1097/WCO.0b013e3283489687 21725242 PMC3154756

[B72] Watson G. (1975). Free agency. J. Philos. 72, 205–220. 10.2307/2024703

